# Correlation of *UGT1A1* genotypes with newborn hyperbilirubinemia using newborn genetic screening

**DOI:** 10.3389/fped.2026.1737797

**Published:** 2026-07-07

**Authors:** Qianyong Ji, Wen Zeng, XiaoOu Li, ShukChing Chong, JianJiang Zhu

**Affiliations:** 1 Department of Obstetrics & Gynaecology, Chinese University of HongKong, Prince of Wales Hospital, Hong Kong SAR, China; 2 Prenatal Diagnosis Center, Haidian District Maternal and Child Health Care Hospital, Beijing, China; 3 Department of Neonatology, Haidian District Maternal and Child Health Care Hospital, Beijing, China

**Keywords:** bilirubin, newborn, newborn genetic screening, newborn hyperbilirubinemia, *UGT1A1* gene

## Abstract

**Background:**

This study evaluated whether uridine diphosphate glucuronosyltransferase 1A1 (*UGT1A1*) variants identified through newborn genetic screening are associated with the development of clinically significant neonatal hyperbilirubinemia.

**Methods:**

Next-generation sequencing (NGS) was performed in 1,019 newborns, and pathogenic (P)/likely pathogenic (LP) *UGT1A1* findings were confirmed by Sanger sequencing. This single-center cohort study was based on routine NBGS and bilirubin assessments, whereas genotype–phenotype correlations were analyzed retrospectively using routinely collected clinical data. Newborns with confirmed biallelic or multi-allelic *UGT1A1* variants were grouped as homozygous (Hom), compound heterozygous (CH, confirmed in trans), or three-loci (3 loci, confirmed in trans), and were compared with a randomly selected control group without *UGT1A1* variants. TcB levels were measured at prespecified timepoints (postnatal days 1, 4, and 42).

**Results:**

Seventy-five newborns had confirmed biallelic or multi-allelic *UGT1A1* variants (Hom, *n* = 45; CH, *n* = 26; 3 loci, *n* = 4), and 40 newborns served as controls. The most frequent variants were c.211G>A and c.-41_-40dup; c.1091C>T and c.686C>A were less common. TcB values did not differ across groups on days 1 and 4, but were higher in all mutation groups than in controls on day 42 (*P* < 0.001), with the highest values observed in the 3-loci group. Clinically significant neonatal hyperbilirubinemia, defined as clinician-documented hyperbilirubinemia requiring phototherapy during the neonatal period, was rare (four cases in total; 3/75 in the mutation group and 1/40 in controls; *P* = 0.367), although the small number of events precludes firm between-group inference.

**Conclusions:**

In this single-center cohort, common *UGT1A1* variants were associated with mildly elevated TcB levels at 6 weeks, suggesting slower postnatal bilirubin clearance, but showed little association with clinically significant neonatal hyperbilirubinemia. These findings support a variant-level reporting strategy for newborn genetic screening (NBGS). Routinely classifying common *UGT1A1* variants (c.211G>A, c.-41_-40dup, c.1091C>T, and c.686C>A) as moderate-/high-risk and performing routine segregation analysis for multi-locus variants would likely increase genetic counseling burden and parental anxiety while providing minimal additional clinical benefit.

## Introduction

Newborn genetic screening (NBGS) is a rapid, simple, and sensitive diagnostic approach used during the neonatal period. It plays a crucial role in detecting congenital and hereditary disorders that may cause physical or neurodevelopmental impairment in children. Early detection enables timely intervention before symptoms appear, thereby preventing irreversible damage and potentially saving lives (1). In addition to traditional biochemical testing for suspected hereditary metabolic diseases, next-generation sequencing (NGS) can be used to screen for monogenic disorders. NGS shortens diagnostic time, predicts disease risk and carrier status, and supports precision medicine. Since 2022, China has promoted and published an expert consensus describing the principles for selecting diseases for NGS-based screening of monogenic disorders (2–4).

Studies of the uridine diphosphate glucuronosyltransferase 1A1 (*UGT1A1*) gene have yielded noteworthy insights. Variants are described according to Human Genome Variation Society (HGVS) nomenclature using the reference transcript *UGT1A1* NM_000463.3. Hereditary nonhemolytic hyperbilirubinemia caused by *UGT1A1* variants includes three clinically defined entities: Crigler–Najjar syndrome type I (CNS-I), Crigler–Najjar syndrome type II (CNS-II), and Gilbert syndrome (GS). Diagnosis is based on serum bilirubin levels and clinical manifestations. CNS-I is characterized by serum bilirubin levels of 20–45 mg/dL. Patients with little or no *UGT1A1* activity may develop bilirubin encephalopathy and may die; survivors often require liver transplantation. CNS-II is characterized by serum bilirubin levels of 6–20 mg/dL with reduced *UGT1A1* activity. Some patients with CNS-II develop severe hyperbilirubinemia with neurological sequelae, whereas others experience only mild symptoms. Phenobarbital and phototherapy can be used to treat CNS-II. GS is associated with serum bilirubin levels of 1–20 mg/dL and usually does not pose a major health threat, although some patients may experience fatigue, mild jaundice, or gastrointestinal symptoms (5–9). Most previous studies have been phenotype-driven and have analyzed gene variants only in visibly jaundiced patients. The present study evaluated *UGT1A1* screening in NBGS by exploring the relationship between genotype and clinical manifestations. We also examined whether *UGT1A1* screening should be included in NBGS.

## Materials and methods

### Ethical approval and consent to participate

This single-center cohort study was approved by the Ethical Committee of the Institute of Haidian District Maternal and Child Health Care Hospital (No. 2024-01). Written informed consent was obtained from the guardians for publication of relevant clinical information for academic purposes.

### Newborns' general information

A total of 1,019 newborns who underwent screening for high-incidence monogenic diseases between July 2022 and November 2023 at Haidian District Maternal and Child Health Care Hospital were selected for analysis (see Table 1). The inclusion criteria were full-term singleton newborns with no structural or chromosomal abnormalities identified prenatally by ultrasound. NBGS was performed in newborns whose guardians provided informed consent to participate in the hospital's NBGS program. On the basis of NBGS results, newborns were categorized into four groups according to the type of *UGT1A1* variants: the homozygous variant group (Hom), the compound heterozygous variant group (CH), the group carrying three alleles (3 loci), and the control group (without *UGT1A1* variants). The control pool consisted of newborns without *UGT1A1* variants who were born between July 2022 and February 2023, yielding a total of 546 newborns. Forty newborns were selected from these 546 using the even-ending random number table method. The present study is a retrospective analysis of prospectively collected routine clinical measurements, including transcutaneous bilirubin (TcB) at prespecified timepoints. Detailed data on several established non-genetic contributors to neonatal hyperbilirubinemia (e.g., feeding type, hemolysis, glucose-6-phosphate dehydrogenase [G6PD] status, and birth trauma) were not uniformly available and therefore were not adjusted for in the analyses.

**Table 1:** Patient demographics.

Group	Number (n)	Sex		Gestational age (d)	Neonate weight (g)
		Male	Female		
Hom	45	21	24	275.8 ± 7.5	3,410.8 ± 347.28
CH	26	14	12	270.9 ± 6.8	3,185.8 ± 376.10
3 loci	4	1	3	266.2 ± 9.3	3,270.0 ± 530.85
Control	40	21	19	276.4 ± 13.9	3,288.0 ± 394.61
*P-value*		0.695		0.051	0.108

Hom, homozygous; CH, compound heterozygosity; 3 loci, newborns carrying three UGT1A1 allelic variants.

### NBGS

NBGS was performed at 3 days of age. Genomic DNA was extracted from dried blood spots prepared from three drops of newborn heel blood using the QIAamp DNA Mini Kit (Qiagen, China). DNA libraries were prepared according to Illumina protocols, including end repair, adapter ligation, and polymerase chain reaction (PCR) enrichment. The amplified DNA was then captured using a whole-exome capture kit (MyGenostics GenCap Enrichment Technologies). Biotinylated capture probes were designed to tile all exons outside repetitive regions. Captured DNA was eluted and amplified for 13 cycles using the following program: 95 °C for 4 min (one cycle); 98 °C for 30 s, 65 °C for 30 s, and 72 °C for 30 s (13 cycles); and 72 °C for 5 min (one cycle). The PCR product was purified using SPRI beads (Beckman Coulter). The enrichment libraries were sequenced on a DNBSEQ-T7 platform with 150-bp paired-end reads. Variant interpretation followed the 2015 ACMG/AMP guidelines for sequence variant classification. Pathogenic (P) and likely pathogenic (LP) *UGT1A1* variants were selected according to these criteria; the corresponding evidence codes and supporting data, including published functional evidence and in-trans parental confirmation when available, are provided.

### Sanger sequencing

Sanger sequencing was used to confirm P/LP variants. Parental blood samples were analyzed to verify the allelic origin. Newborns with variants confirmed in trans were included in the mutation group as compound heterozygotes. Hom variants were included in the mutation group without additional phase confirmation.

### Comparison of neonatal TcB values

TcB values on postnatal days 1, 4, and 42 were measured using the JH20-1C transcutaneous jaundice detector (Nanjing University of Science and Technology Technology Development Co., Ltd.; Registration No. Su Xie Zhu Zhun 20162221152) (Table 2).

**Table 2:** Tcb values.

Group	Day 1 (mg/dL)	Day 4 (mg/dL)	Day 42 (mg/dL)
Hom (*n* = 45)	5.9 ± 3.0	12.0 ± 1.6	5.5 ± 2.3
	(2.2-17.1)	(7.8–15.7)	(1.1–10.9)
CH (*n* = 26)	5.0 ± 1.0	12.9 ± 1.7	6.1 ± 2.2
	(3.0–7.5)	(8.7–15.7)	(2.8–9.7)
3 loci (*n* = 4)	5.7 ± 1.9	13.2 ± 0.6	8.6 ± 2.4
	(3.2–7.5)	(12.3–13.6)	(5.8–11.3)
Control (*n* = 40)	5.1 ± 1.3	12.7 ± 2.2	4.2 ± 2.3
	(1.4–7.5)	(4.6–16.8)	(0.8–10.2)

TcB, transcutaneous bilirubin. Values are presented as mean ± SD (range).

The scheduling of TcB measurements on postnatal days 1, 4, and 42 was designed on the basis of the physiological progression of neonatal jaundice and established clinical practice. Day 1 measurements provide an early baseline and help identify rapid bilirubin increases that may indicate hemolytic disease or other early-onset pathological conditions (10). Day 4 corresponds to the critical window for predischarge or early post-discharge risk assessment, because serum bilirubin typically peaks between 72 and 96 h of age in term and late-preterm newborns (11). Day 42 was selected because it corresponds to the routine postnatal follow-up visit in China (12). Assessment at this timepoint is useful for documenting the resolution of visible jaundice and identifying prolonged hyperbilirubinemia, such as breast milk jaundice or delayed bilirubin clearance potentially influenced by *UGT1A1* genetic variants, after the initial physiological phase has passed (13).

### Outcome definitions and post-positive clinical workflow

For outcome classification, routine clinical records were reviewed together with available total serum bilirubin results. Clinically significant neonatal hyperbilirubinemia was operationally defined as clinician-documented jaundice requiring phototherapy during the neonatal period. TcB was used as a screening measure and was not used alone to define severe hyperbilirubinemia. Physiologic jaundice was defined as jaundice appearing after the first 24 h of life, managed without phototherapy or exchange transfusion, showing spontaneous improvement on routine follow-up, and, when serum bilirubin was measured, remaining below the level considered to require treatment in routine care. Serum bilirubin testing was obtained only when clinically indicated by the treating neonatologist and was not performed uniformly in all newborns with elevated TcB values. Clinical decisions regarding serum bilirubin testing and initiation of phototherapy were made as part of routine neonatal care rather than according to a study-mandated standardized bilirubin threshold. Therefore, the outcome comparison in this study was based primarily on clinical management categories rather than on uniform serum bilirubin-based biochemical classification for the entire cohort.

After an NBGS-positive *UGT1A1* finding, the routine workflow included disclosure of the result to guardians, confirmatory Sanger sequencing, parental testing for segregation analysis when multi-allelic findings were suspected, and routine clinical follow-up based on the neonatologist's assessment. This workflow increased counseling time and family communication workload, particularly for common variant combinations with uncertain immediate clinical impact.

### Statistical methods

SPSS (version 26.0) was used for statistical analyses. Categorical variables were summarized as counts and percentages and compared using the chi-square test; Fisher's exact test was used when expected cell counts were small. Continuous variables were summarized as mean ± standard deviation (mean ± SD). One-way analysis of variance (ANOVA) was used for overall comparisons of TcB values across the four groups, and pairwise between-group comparisons were performed for prespecified contrasts (Table 3).

**Table 3:** Comparison of TcB values.

Comparison	Day 1	Day 4	Day 42
Four groups	*P* = 0.257	*P* = 0.195	***P*** **=** **0.000227**
Hom−Control	*P* = 0.084	*P* = 0.105	***P*** **=** **0.011**
CH−Control	*P* = 0.926	*P* = 0.719	***P*** **=** **0.002**
3 loci−Control	*P* = 0.604	*P* = 0.623	***P*** **=** **0.000319**
Hom−CH			*P* = 0.294
Hom−3 loci			***P*** **=** **0.009**
CH−3 loci			***P*** **=** **0.038**

The *P* values in the “Four groups” row represent overall between-group comparisons (one-way ANOVA), whereas the remaining *P* values represent pairwise comparisons.

Bold values indicate statistically significant differences (*P* < 0.05).

Because the 3-loci group was very small (*n* = 4), ANOVA-based results involving this group should be interpreted as exploratory; this limitation is acknowledged. The significance level was set at *α* = 0.05, and *P*-values <0.05 were considered statistically significant. No formal *a priori* sample size calculation was performed because this was an exploratory single-center study based on all eligible NBGS participants during the study period and the available follow-up cohort.

## Results

### Comparison of general information of newborns in four groups

**Figure 1:**
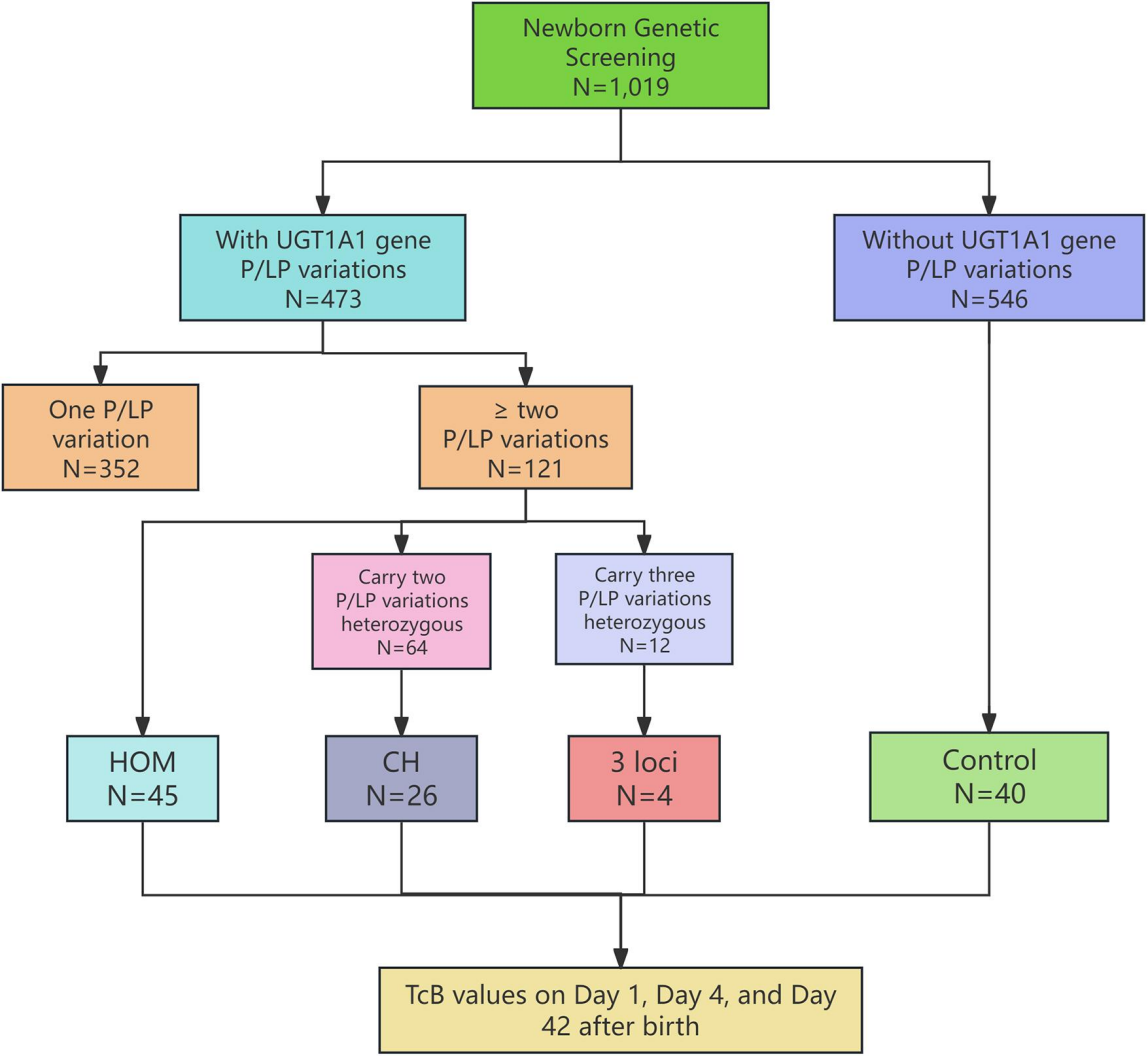
Consort diagram.

Our study included 1,019 newborns. In the final dataset used for grouping (Figure 1), 546 newborns had no detected *UGT1A1* variants and 473 had at least one *UGT1A1* variant. Of these, 121 had two or more P/LP allelic variants. Among these 121 newborns, 45 were Hom, 64 had two heterozygous variants (including 26 confirmed in trans), and 12 had three variants (including four confirmed in trans). Accordingly, the Hom group contained 45 cases, the CH group 26 cases, and the 3-loci group four cases (see Figure 1). The NBGS-positive rate was 75/1,019 (7.4%). Forty newborns without *UGT1A1* variants were randomly selected as controls from the 546 newborns without detected *UGT1A1* variants using the even-ending random number table method. The four groups did not differ significantly in sex, gestational age, or birth weight (*P*-values > 0.05; see Table 1).

### LP *UGT1A1* variants

Four LP variants classified according to the American College of Medical Genetics and Genomics (ACMG) guidelines (14) were identified in the mutation groups: c.211G>A, c.-41_-40dup, c.1091C>T, and c.686C>A. The allele frequencies were 9.7% for c.211G>A, 1.8% for c.-41_-40dup, 0.3% for c.1091C>T, and 0.5% for c.686C>A. The carrier rates (*n* = 352) were 59.9% for c.211G>A, 28.1% for c.-41_-40dup, 9.9% for c.1091C>T, and 0.6% for c.686C>A. The corresponding East Asian population frequencies in gnomAD were 0.153 for c.211G>A, 0.12210 for c.-41_-40dup, 0.01168 for c.1091C>T, and 0.01949 for c.686C>A (15–18) (see Table 4).

**Table 4:** Detected UGT1A1 variants with allele frequencies and carrier rates

Variants	Amino acid	ACMG	Evidence	Allele frequency (overall, *n* = 1,019)	Allele frequency (three variant groups, *n* = 75)	gnomAD EAS allele frequency	Carrier rate (carriers, *n* = 352)
c.211G>A	p.G71R	LP	PM3_Strong, PS3_Moderate	100/2,038 (9.7%)	100/150 (66.7%)	0.1530	211/352 (59.9%)
c.-41_-40dup	p.?	LP	PM3_Strong, PS3_Moderate	37/2,038 (1.8%)	37/150 (24.7%)	0.12210	99/352 (28.1%)
c.1091C>T	p.P364L	LP	PM3_Strong, PS3_Moderate	7/2,038 (0.3%)	7/150 (4.7%)	0.01168	35/352 (9.9%)
c.686C>A	p.P229Q	LP	PS3_Moderate, PM3_Moderate,PM2_Supporting	10/2,038 (0.5%)	10/150 (6.7%)	0.01949	2/352 (0.6%)
c.1456T>G	p.Y86D	LP	PM3_Strong, PP3_Moderate, PM2_Supporting	—	Not detected (*n* = 75 group)	—	5/352 (1.4%)

ACMG, American College of Medical Genetics and Genomics Guidelines; LP, likely pathogenic; PM3_Strong, for recessive disorders, detected in-trans with a pathogenic or likely pathogenic variant in an affected patient; PS3 moderate, well-established *in vitro* or *in vivo* functional studies supportive of a damaging effect on the gene or gene product; PM3_Moderate, for recessive disorders, the variant is detected in trans with a pathogenic or likely pathogenic variant in affected individual(s), providing moderate allelic evidence; PM2_Supporting, the variant is absent, or present at an extremely low frequency, in large population databases consistent with the prevalence and penetrance of the disorder, providing supporting-level evidence for rarity; PP3_Moderate, multiple validated computational prediction tools and/or conservation analyses provide concordant evidence supporting a deleterious effect on the gene or gene product, with sufficient weight to be considered moderate-level evidence.

### Day-42 TcB levels across four groups

**Figure 2:**
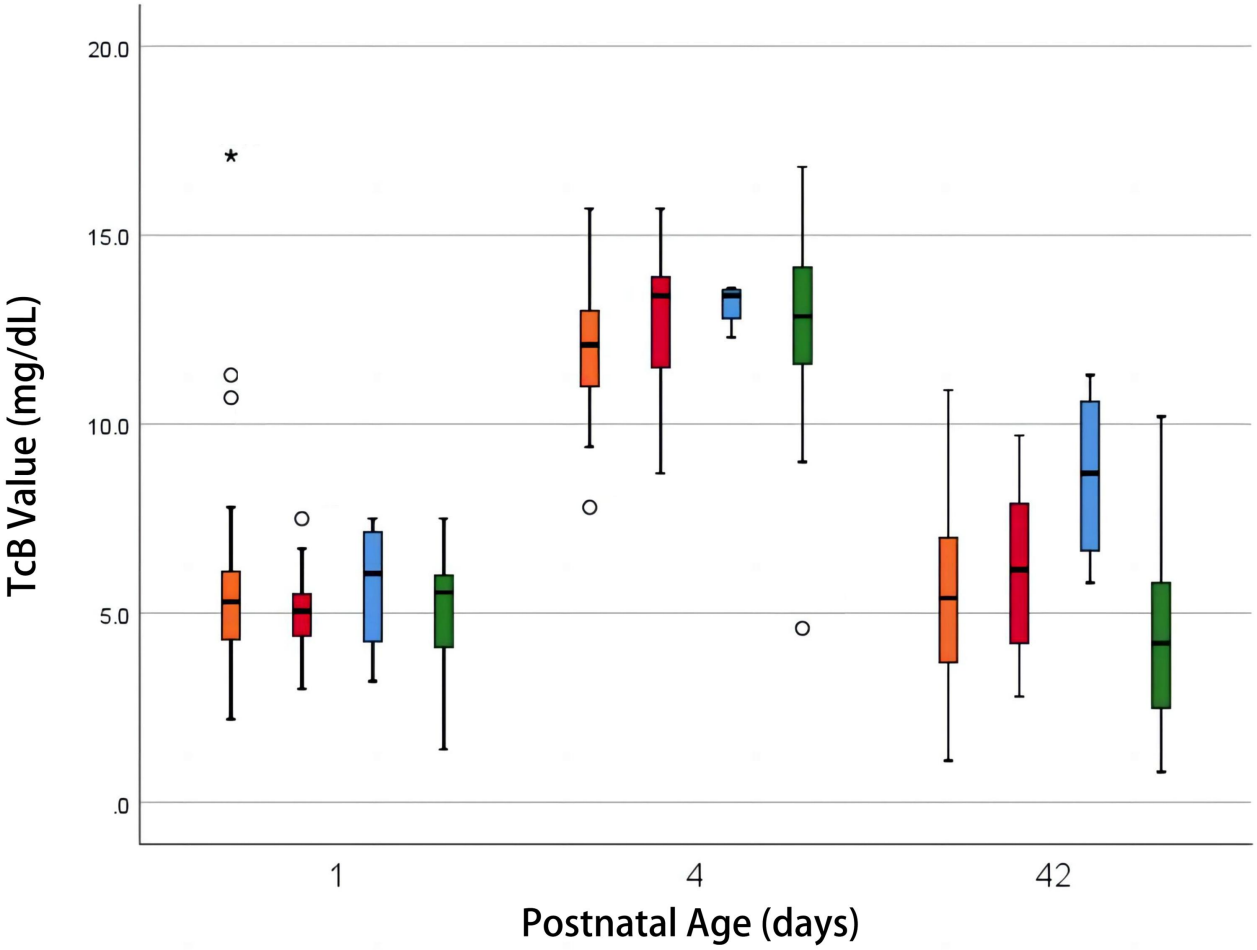
Boxplot of transcutaneous bilirubin (TcB) values (mg/dL) across the four groups. The *x*-axis shows postnatal age (days 1, 4, and 42), and the *y*-axis shows TcB values (mg/dL). Colors indicate groups: Hom (orange), CH (red), 3 loci (blue), and Control (green). Boxes represent the interquartile range (IQR, 25th–75th percentile), and the thick horizontal line indicates the median. Whiskers extend to the smallest and largest values within 1.5 times the IQR. Outliers (circles) are defined as values between 1.5 and 3 times the IQR from the edge of the box, whereas extreme outliers (asterisks) are values more than 3 times the IQR beyond the box edge.

TcB values on postnatal days 1, 4, and 42 were compared across the four groups. No statistically significant differences were observed on days 1 and 4 (*P*-values > 0.05; Figure 2 and Table 3). Although no significant between-group difference was observed on day 4, mean TcB values across groups ranged from 12.0 to 13.2 mg/dL (Table 2). These day-4 TcB values indicate that some infants were within a clinically relevant screening range; however, because confirmatory serum bilirubin testing was not performed uniformly in all infants with elevated TcB values, these TcB findings should not be interpreted as direct biochemical evidence of treatment-threshold or severe hyperbilirubinemia. By contrast, TcB values differed significantly across the four groups on day 42. In Table 3, the *P*-value in the “Four groups” row represents the overall between-group comparison, whereas the remaining *P* values represent pairwise comparisons. Compared with controls, all three mutation groups had higher day-42 TcB values (*P*-values <0.05). Pairwise comparisons among the mutation groups showed that the mean day-42 TcB values in the Hom group (5.5 ± 2.3 mg/dL) and CH group (6.1 ± 2.2 mg/dL) were lower than that in the 3-loci group (8.6 ± 2.5 mg/dL) (Table 2). Given the small sample size of the 3-loci group (*n* = 4), these findings should be interpreted cautiously.

### Day-42 TcB levels by *UGT1A1* genotype

Seven *UGT1A1* genotypes identified by Sanger sequencing were analyzed to explore the association between genotype and day-42 TcB value. No statistically significant association between day-42 TcB value and genotype category was observed (*P*-value >0.05; Table 5). Likewise, no statistically significant difference in day-42 TcB value was observed between the Hom and CH groups (*P*-value >0.05).

**Table 5:** Tcb values at day 42.

Genotype group	Genotype		Genotype frequency	TcB values (mg/dL)	
	Allele 1	Allele 2			
Hom	c.211G>A	c.211G>A	36/45	5.54 ± 2.40	*P* = 0.472
	c.-41_-40dup	c.-41_-40dup	7/45	5.17 ± 1.83	
	c.1091C>T	c.1091C>T	2/45	7.40 ± 0.71	
CH	c.211G>A	c.-41_-40dup	17/26	5.70 ± 2.19	*P* = 0.492
	c.686C>A	c.-41_-40dup	6/26	6.82 ± 2.43	
	c.211G>A	c.1091C>T	3/26	6.73 ± 1.52	
3 loci	c.[211G>A;686C>A]	c.211G>A	4/4	8.63 ± 2.45	
*P* = 0.131					

### Clinical neonatal jaundice outcomes

Three newborns in the mutation groups and one in the control group developed clinically significant neonatal hyperbilirubinemia requiring phototherapy. The remaining newborns were managed without phototherapy and were clinically consistent with physiologic jaundice. Serum bilirubin results were reviewed when available, but serum testing was not performed uniformly in all non-phototherapy cases. Likewise, not all newborns with elevated TcB values underwent confirmatory laboratory testing. Therefore, Table 6 presents clinical neonatal jaundice outcome categories based on actual clinical management rather than uniform serum bilirubin-confirmed classifications across the entire cohort. Because only four phototherapy cases occurred overall (3/75 vs. 1/40), this between-group comparison should be interpreted descriptively and cautiously (Fisher's exact *P* = 0.367).

**Table 6:** Comparison of clinical neonatal jaundice outcomes between mutation and control groups.

Clinical outcome	Mutation	Control
Significant hyperbilirubinemia	3	1
No phototherapy	72	39
Total	75	40
*P* = *0.367*		

No phototherapy, newborns clinically consistent with physiologic jaundice and managed without phototherapy.

*P* value calculated using Fisher's exact test. Because only four phototherapy cases occurred overall, this comparison should be interpreted descriptively and cautiously.

## Discussion

UGTs are a family of isoenzymes found primarily in hepatocytes. *UGT1A1* is the major bilirubin-conjugating isoenzyme and plays a central role in bilirubin glucuronidation and biliary excretion (19). *UGT1A1* is located on chromosome 2q37.1, and it contains five exons (20). Variants in the coding region of *UGT1A1* reduce enzyme activity or abolish *UGT1A1* function, leading to inherited unconjugated hyperbilirubinemia syndromes such as CNS-I, CNS-II, and GS. Previous studies have shown that severe variants are more often associated with marked loss of activity and severe phenotypes, whereas many missense variants are associated with milder phenotypes such as GS or some CNS-II presentation (21).

The variants detected in the three mutation groups in this study were c.211G>A, c.-41_-40dup, c.1091C>T, and c.686C>A. Among them, c.211G>A is a well-recognized hotspot variant associated with *UGT1A1*-related hyperbilirubinemia in East Asian populations, whereas the other three are also relatively common alleles in this population (15–18, 22–24). Functional studies have shown that homozygous or compound heterozygous *UGT1A1* variants can substantially reduce enzyme activity, although the degree of reduction and the clinical expression vary across genotypes (25–27). A Japanese study examining the association between 163 patients with CNS-II and GS and different *UGT1A1* genotypes found that all genotypes observed in patients with GS were also detected in the present study (14, 23). Importantly, however, our study differed from most prior reports in that it was NBGS-based and included predominantly asymptomatic newborns identified before severe disease presentation. This distinction is important because variants that are classified as pathogenic or likely pathogenic in a disease-oriented recessive framework do not necessarily confer a high risk of clinically significant neonatal hyperbilirubinemia when detected incidentally through population newborn screening.

A key finding of the present study was that TcB values did not differ significantly among groups on postnatal days 1 and 4, but were significantly higher in all three mutation groups than in controls on day 42. In the current cohort, mean day-4 TcB values ranged from 12.0 to 13.2 mg/dL across groups, whereas mean day-42 TcB values ranged from 4.2 mg/dL in controls to 8.6 mg/dL in the 3-loci group. These findings suggest that common *UGT1A1* hotspot variants may be associated more strongly with delayed bilirubin clearance or prolonged mild jaundice than with marked early neonatal bilirubin elevation. This interpretation is biologically plausible because reduced *UGT1A1* activity can slow bilirubin conjugation and contribute to prolonged unconjugated hyperbilirubinemia, including persistent jaundice phenotypes reported in Asian populations (6, 28–30). In addition, the higher day-42 TcB levels observed in newborns carrying three *UGT1A1* allelic variants than in the biallelic variant groups suggest that increasing allelic burden may be associated with slower bilirubin clearance, although this finding should be interpreted cautiously given the very small sample size of the 3-loci group.

These TcB findings should nevertheless be interpreted cautiously. Although TcB is a useful noninvasive screening tool, it is not interchangeable with TSB, especially when bilirubin levels are higher and treatment decisions are being considered (11). In our cohort, mean day-4 TcB values ranged from 12.0 to 13.2 mg/dL across groups (see Table 2), indicating that some infants were in a clinically relevant screening range. Therefore, these day-4 TcB values should not be interpreted as direct evidence of treatment-threshold hyperbilirubinemia in the absence of corresponding serum bilirubin measurements. In addition, confirmatory serum bilirubin testing was not performed uniformly in all infants with elevated TcB values, including those with TcB values above 12.5 mg/dL. Therefore, TcB values in this range should be interpreted as screening findings rather than direct biochemical evidence of severe or treatment-threshold hyperbilirubinemia. Rather, they indicate that some infants entered a clinically relevant screening range, whereas the more consistent genotype-associated signal in this cohort was the higher TcB level at day 42, suggesting slower postnatal bilirubin clearance rather than severe early neonatal hyperbilirubinemia. In this study, clinically significant neonatal hyperbilirubinemia was operationally defined primarily on the basis of actual clinical management, specifically the requirement for phototherapy, rather than on the basis of a uniform TcB cutoff or uniformly available serum bilirubin threshold across the cohort. Because this was a retrospective study based on routine clinical care, the recognition of visible jaundice and the decision to obtain serum bilirubin testing or initiate phototherapy may have varied across clinicians, which could have resulted in under-ascertainment of some infants with clinically relevant jaundice or elevated bilirubin levels.

Consistent with this interpretation, clinically significant neonatal hyperbilirubinemia was uncommon in our cohort, with only four cases overall (3/75 vs. 1/40). Because of the very small number of events, between-group comparisons should be interpreted cautiously. Taken together, these findings suggest that the common *UGT1A1* variants detected through NBGS may have relatively low penetrance for clinically significant neonatal hyperbilirubinemia during the neonatal period, despite remaining relevant diagnostic variants in infants and children with persistent unconjugated hyperbilirubinemia or suspected inherited bilirubin disorders (14, 21, 23). In this context, some common *UGT1A1* variants may act mainly as low- to moderate-effect risk alleles or phenotype modifiers rather than as strong predictors of clinically significant neonatal hyperbilirubinemia when identified through population screening.

These findings also have implications for NBGS reporting strategy. NBGS should balance disease severity, prevalence, penetrance, and the availability of effective early intervention when determining which findings should be routinely returned to families (2, 4, 31). For *UGT1A1*, routinely reporting common GS/CNS-II-associated hotspot combinations may offer limited immediate neonatal benefit while increasing confirmatory testing, segregation analysis, parental anxiety, and genetic counseling workload. A more selective variant-level reporting strategy focused on findings with clearer and more actionable neonatal risk may better preserve clinical benefit while reducing unnecessary burden on families and healthcare services (2, 4, 31).

Several limitations should also be considered when interpreting these findings. This was a single-center study from one geographic region, and the 3-loci subgroup was very small; therefore, comparisons involving this subgroup should be interpreted cautiously. In addition, bilirubin phenotyping relied primarily on TcB at prespecified timepoints, whereas serum bilirubin testing was obtained according to clinical indication rather than uniformly in all newborns. Several important non-genetic contributors to neonatal hyperbilirubinemia were also not consistently available for adjustment. Accordingly, larger multicenter studies using standardized serum bilirubin endpoints and more complete clinical covariates will be important to refine the penetrance, short-term clinical relevance, and reporting threshold of *UGT1A1* findings in NBGS.

### Limitations

This study has several limitations. First, although bilirubin measurements were collected as part of routine clinical care at prespecified timepoints, the present genotype–phenotype analysis was retrospective and conducted at a single center, which may limit generalizability and introduce selection or information bias. Second, no formal *a priori* sample size calculation was performed because this was an exploratory study based on all eligible NBGS participants during the study period and the available follow-up cohort; therefore, the statistical power to detect small between-group differences may be limited. This concern is particularly relevant for the 3-loci group (*n* = 4), and comparisons involving this subgroup should be interpreted cautiously. Third, bilirubin testing relied primarily on TcB at prespecified timepoints. Although TcB is useful for noninvasive screening, it is not interchangeable with TSB, especially when bilirubin levels are higher and treatment decisions are being considered (11). Serum total bilirubin and conjugated/direct bilirubin data were not uniformly available, which limited biochemical phenotyping and precluded direct interpretation of elevated TcB values as treatment-threshold hyperbilirubinemia. Fourth, several important non-genetic contributors to neonatal hyperbilirubinemia (e.g., feeding pattern, dehydration, hemolysis, G6PD deficiency, and birth trauma) were not uniformly collected and could not be adjusted for in the statistical analyses. No physician-verified long-term outcome assessment was available in this study. Fifth, because this was a retrospective study based on routine clinical care, the exact number of clinicians involved in jaundice assessment and the inter-observer consistency in recognizing visible jaundice were not available. Therefore, ascertainment of jaundice, serum bilirubin testing, and phototherapy initiation may have varied across clinicians, and some infants with clinically relevant jaundice or elevated bilirubin levels may have been under-recognized.

## Conclusion

NBGS should balance disease severity, prevalence, penetrance, and the availability of effective early interventions (2, 4, 31) while moving toward variant-level reporting criteria that specify which variants or genotypes warrant disclosure to families. In this single-center cohort, common *UGT1A1* variants were associated with modestly higher TcB levels at 6 weeks but were rarely accompanied by clinically significant neonatal hyperbilirubinemia. These results indicate that classifying common *UGT1A1* variants (c.211G>A, c.-41_-40dup, c.1091C>T, and c.686C>A) as reportable moderate-/high-risk findings—and routinely pursuing segregation confirmation for multi-locus patterns—may add substantial counseling demand and heighten parental distress, with little additional clinical yield. These conclusions should be interpreted in light of the exploratory design, the small multi-locus subgroup size, and the use of TcB-based rather than serum bilirubin-based testing. Larger multicenter studies using serum bilirubin and standardized endpoints are needed to refine reporting and follow-up strategies for *UGT1A1* in NBGS.

## Data Availability

The original contributions presented in the study are publicly available. This data can be found here: https://datadryad.org/dataset/doi:10.5061/dryad.6m905qgft.
